# Effects of L-Citrulline Supplementation and Aerobic Training on Vascular Function in Individuals with Obesity across the Lifespan

**DOI:** 10.3390/nu13092991

**Published:** 2021-08-27

**Authors:** Anaisa Genoveva Flores-Ramírez, Verónica Ivette Tovar-Villegas, Arun Maharaj, Ma Eugenia Garay-Sevilla, Arturo Figueroa

**Affiliations:** 1 Department of Medical Science, Division of Health Science, University of Guanajuato, Campus León, León 37320, Mexico; Anaisa_390@hotmail.com (A.G.F.-R.); Veronicatovar92@hotmail.com (V.I.T.-V.); 2 Department of Kinesiology and Sport Management, Texas Tech University, Lubbock, TX 79409, USA; arun.maharaj@ttu.edu

**Keywords:** obesity, children, vascular function, aerobic training, L-Citrulline, L-Arginine, nitric oxide

## Abstract

Children with obesity are at higher risk for developing cardiometabolic diseases that once were considered health conditions of adults. Obesity is commonly associated with cardiometabolic risk factors such as dyslipidemia, hyperglycemia, hyperinsulinemia and hypertension that contribute to the development of endothelial dysfunction. Endothelial dysfunction, characterized by reduced nitric oxide (NO) production, precedes vascular abnormalities including atherosclerosis and arterial stiffness. Thus, early detection and treatment of cardiometabolic risk factors are necessary to prevent deleterious vascular consequences of obesity at an early age. Non-pharmacological interventions including L-Citrulline (L-Cit) supplementation and aerobic training stimulate endothelial NO mediated vasodilation, leading to improvements in organ perfusion, blood pressure, arterial stiffness, atherosclerosis and metabolic health (glucose control and lipid profile). Few studies suggest that the combination of L-Cit supplementation and exercise training can be an effective strategy to counteract the adverse effects of obesity on vascular function in older adults. Therefore, this review examined the efficacy of L-Cit supplementation and aerobic training interventions on vascular and metabolic parameters in obese individuals.

## 1. Introduction

Overweight and obesity are defined as abnormal or excessive fat accumulation [1]. In adults, the World Health Organization (WHO) defines obesity as a Body Mass Index (BMI) greater than or equal to 30 kg/m^2^, and for children aged between 5–19 years, obesity is considered two standard deviations above the WHO Growth Reference median [1]. Approximately 340 million children and adolescents worldwide were classified as overweight or obese in 2016 and the prevalence is dramatically increasing [1,2]. The prevalence of hypertension is greater than 70% and increases with progression of obesity grade in adults [3]. In obese children, the prevalence of hypertension is 15.27%, which is substantially higher than 1.9% in those with normal weight [4]. Obesity is a risk factor for the development of cardiovascular diseases (CVD) and type 2 diabetes (T2D) due to the association with hypertension and insulin resistance [1].

In some cases, individuals are classified as obese based on BMI alone but considered “metabolically healthy obese” (MHO) [5] since they display a normal cardiometabolic profile such as optimal insulin sensitivity, blood pressure, lipid and inflammatory profiles [6]. However, although MHO individuals are relatively protected against cardiometabolic diseases compared to metabolically unhealthy obese, MHO should not be considered a harmless condition as they have a higher risk of developing obesity-related diseases compared to normal weight individuals [6,7,8].

Obesity is a condition strongly associated with metabolic syndrome (MetS), defined as a constellation of physiological, biochemical, clinical and metabolic factors that are associated with an increased risk of atherosclerosis, T2D and all-cause mortality [9]. MetS can be diagnosed in children (10 to 16 years old) with abdominal obesity and at least two clinical features such as elevated triglycerides, low levels of high-density lipoprotein (HDL) cholesterol, high blood pressure (hypertension) and high fasting blood glucose (hyperglycemia) [10,11,12]. Childhood obesity and hypertension predict MetS later in life [13,14]. In children, hypertension is a prevalent cardiovascular risk factor associated with reduced endothelial function, increased vascular thickness and arterial stiffness [15]. A hallmark risk factor of MetS is insulin resistance (IR) [16], which is an impairment of insulin function to promote glucose uptake in insulin-sensitive target tissues, such as skeletal muscle and adipose tissue [17], resulting in abnormal glucose homeostasis [16]. Fasting serum glucose values between 86 and 99 mg/dL during childhood increase the risk of developing T2D during adulthood twofold [18], highlighting the importance of identifying and developing treatment strategies to prevent adult-onset of metabolic complications in obese children and adolescents.

**Figure 1:**
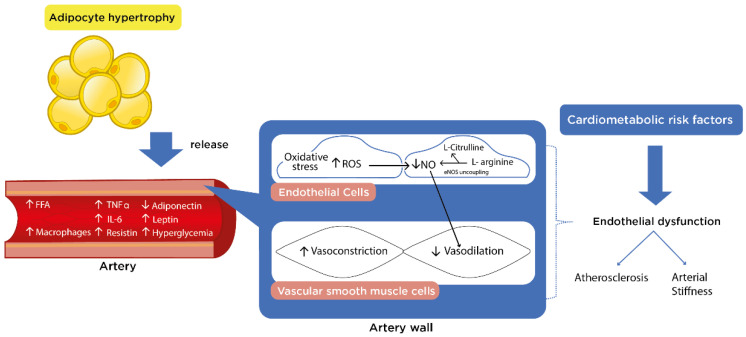
Obesity and endothelial dysfunction. Adipocyte hypertrophy leads to release of FFA, leptin, resistin, TNFα and IL-6 into the vascular wall, promoting inflammation, while anti-inflammatory adiponectin secretion is reduced. Proinflammatory adipokines and hyperglycemia induce the production of ROS, which by uncoupling eNOS leads to reduced NO synthesis and bioavailability for vasodilation, promoting a vasoconstrictor state. Cardiometabolic risk factors contribute to endothelial dysfunction, characterized by a reduced NO bioavailability, which promotes atherosclerosis and arterial stiffness and development of CVD. FFA: Free fatty acids; TNF-α: Tumor necrosis factor alpha; IL-6: Interleukin-6; ROS: Reactive oxygen species; NO: Nitric oxide; eNOS: endothelial NO synthase; CVD: Cardiovascular disease; ↑: Increase; ↓: Decrease.

Obesity is also associated with elevated levels of proinflammatory adipokines released by visceral adipose tissue that contribute to the development of IR and impaired endothelial function [19]. Endothelial dysfunction is the result of prolonged hyperglycemia, damaging vascular function and structure that eventually leads to CVD development [19]. Proinflammatory adipokines increase the production of reactive oxygen species (ROS) which triggers the release of inflammatory cytokines, adhesion molecules and growth factors that promote cellular oxidative stress [20]. Oxidative stress causes endothelial dysfunction, characterized by a reduction in nitric oxide (NO) and an increase in endothelium-derived vasoconstrictors such as endothelin-1 [19,20] (Figure 1).

For these reasons, it is important to evaluate interventions to improve vascular and metabolic function in obese individuals. There are non-pharmacological treatments that can improve the cardiometabolic profile. L-Citrulline (L-Cit) is a non-essential amino acid not used for protein synthesis, but with a key regulatory role of nitrogen homeostasis [21]. Studies in humans have demonstrated the effect of L-Cit supplementation on improving nitrogen homeostasis and its ability to increase the L-Arginine-NO pathway [21]. In middle-aged adults, oral L-Cit supplementation has shown to improve endothelial function [22], blood pressure [23,24] and arterial stiffness [25] through stimulation of the L-Arginine-NO pathway which consequently leads to vasodilation [23,26]. L-Cit supplementation has also improved lean mass and reduce fat mass in malnourished older adults [27].

The development of childhood obesity is associated with sedentary behavior [28], and increased physical activity is recommended to improve overall health in children with excess adiposity [29,30]. In children and adolescents, aerobic training helps to improve blood pressure [31,32], endothelial function [32,33], arterial stiffness [31,32] atherosclerosis [32], lipid profile [32,33,34,35] and body composition [31,33,35]. The use of L-Cit plus exercise, in middle-aged and older adults with obesity-related diseases or risks factors, has yielded improvements in systolic blood pressure (SBP), pressure wave reflection and aortic stiffness [23,25]. These lifestyle and dietary interventions were implemented in middle-aged and older adults and have elicited no harmful effects. However, there is a void in the literature regarding the efficacy of L-Cit supplementation with and without exercise training in children and adolescents. Thus, the objective of this review is to discuss the effects of L-Cit supplementation and aerobic training interventions on vascular and metabolic parameters in middle-aged and older adults, and deliberate possible avenues of research surrounding similar interventions in obese children and adolescents by observing how obesity can lead to these cardiometabolic alterations.

## 2. Endothelial Function

The endothelium is a layer of cells between the vessel lumen and the vascular smooth muscle cells (VSMC). The most important vasodilator produced by the endothelium is NO, generated from L-Arginine (L-Arg) by endothelial-NO synthase (eNOS) [36]. NO diffuses into the VSMC where it stimulates soluble guanylyl cyclase and subsequently activates cyclic guanosine monophosphate, leading to a decrease in intracellular calcium concentrations, and therefore, to relaxation and vasodilation. NO is considered an anti-atherogenic agent and prevents platelet aggregation, smooth cell proliferation and adhesion of leukocytes to the endothelium [37]. Therefore, vascular homeostasis depends on NO bioavailability.

Endothelial dysfunction is a reversible pathological complication derived from reduced NO bioavailability and impaired vasodilation [38]. Inflammation, oxidative stress, hypertension, dyslipidemia and IR are the main contributing factors in obesity-related endothelial dysfunction [19] through the unbalance between increased ROS and reduced antioxidant capacity [37]. ROS reduces levels of tetrahydrobiopterin (BH_4_), an essential cofactor for eNOS [37], by inducing BH_4_ oxidation (BH_4_ to BH_2_) which leads to eNOS uncoupling [39]. In obesity, a main mechanism for endothelial dysfunction is eNOS uncoupling due to reduced L-Arg bioavailability and BH_4_ oxidation [38,40], leading to less NO bioavailability and increased ROS (superoxide anion and peroxynitrite) generation [36]. Enhanced oxidative stress by ROS upregulates arginase activity/expression competing with eNOS for L-Arg, a common substrate. Cardiometabolic risk factors (obesity, hyperglycemia, hypertension) stimulates arginase to contribute to further ROS production [41,42,43]. Arginase converts L-Arg to L-ornithine and urea, decreasing L-Arg availability for eNOS. Evidence has demonstrated that obesity-induced endothelial dysfunction associated with arterial stiffening, hyperglycemia, hypertension, and oxidative stress were prevented with arginase inhibition [43]. Thus, obesity-induced endothelial dysfunction may be reversible by therapies that increase L-Arg bioavailability and induce arginase inhibition [38,40].

Under normal conditions, insulin favors the release of NO by activation of eNOS, and therefore, has vasodilator, anti-inflammatory and anti-atherosclerotic effects [44]. Hyperinsulinemia contributes to increased vasoconstriction through mitogen activated protein kinase signaling by releasing endothelin-1, a powerful vasoconstrictor agent that promotes IR, oxidative stress and reduced NO bioavailability [44,45]. These responses stimulate the production of pro-inflammatory interleukins which facilitates the progression of vascular wall inflammation [19,46].

In a healthy individual, leptin inhibits insulin production in pancreatic β cells, while insulin stimulates leptin production in adipocytes. In a state of leptin resistance, characterized by hyperleptinemia, leptin ceases the inhibition of insulin production leading to a phase of hyperinsulinemia and IR [47]. Moreover, elevated leptin in obesity contributes to increase blood pressure through increased renal sympathetic activity [48] and oxidative stress in VSMC, reducing vasodilation [49]. Leptin and adiponectin have antagonistic effects on vascular tone regulation, inducing vasoconstriction and vasodilation, respectively [45]. Adiponectin promotes glucose metabolism and fatty acid oxidation, contributes to lower IR [50], and may protect against hypertension through an endothelial-dependent mechanism [48]. Hypoadiponectinemia in obesity is associated with increased leptin [45], IR, impaired glucose and fat metabolism, and consequently, hyperglycemia and increased fat accumulation [50]. In summary, obesity triggers a series of cardiometabolic risk factors that can lead to endothelial dysfunction, a complication characterized by NO reduction that may be reversible with therapies that promote NO production.

### Endothelial-Mediated Vasodilation

Flow-mediated vasodilation (FMD) is a non-invasive technique commonly used to assess macrovascular endothelial function [15]. FMD evaluates the capacity of conduit arteries (e.g., brachial, femoral, popliteal) to increase their diameter relative to the baseline diameter in response to transient ischemia induced by 5 min of arterial occlusion [15]. Brachial artery FMD is considered the gold standard non-invasive measure of endothelial function and is a predictor of CVD [15]. The increase in arterial diameter indicates the vasodilator effect derived from local production of NO induced by increased shear stress after rapid reperfusion. Impaired FMD is associated with atherosclerosis and arterial stiffness [51] and is apparent in children and adolescents with chronic kidney disease [52], T2D and type 1 diabetes mellitus [53]. It has been shown that children with obesity have a lower FMD than normal-weight counterparts [33]. FMD may be a useful tool to utilize and identify early vascular dysfunction in children and young adults with obesity, as many of them may not show clinical manifestations or cardiometabolic risk factors [54].

Middle-aged adults with prediabetes showed endothelial dysfunction and increased oxidative stress [55]. In children and adolescents, endothelial dysfunction assessed as brachial artery FMD was inversely related to age, total and abdominal obesity, blood pressure, fasting insulin and glucose, and homeostatic model assessment-insulin resistance (HOMA-IR) [56,57,58,59]. IR impairs endothelial function even in children and adolescents [57]. Hyperglycemia increases the production of ROS and activity of arginase 1, which mediates endothelial dysfunction by decreasing L-Arg bioavailability [60]. To sum up, cardiometabolic risk factors are associated with endothelial dysfunction, and obese children and adolescents may present lower brachial artery FMD compared to lean counterparts; therefore, this is a useful technique to evaluate the cardiovascular risk in the obese pediatric population.

## 3. Vascular Function and Structure in Individuals with Obesity

Obesity fosters a pro-inflammatory milieu primarily due to abnormally high visceral adipose tissue [9] leading to low-grade chronic inflammation, oxidative stress [61], IR, and impaired endothelial function [62]. Together, this collection of risk factors, if left untreated, may lead to the development of hypertension, atherosclerosis, arterial stiffening [15,52,53], and ultimately, CVD and T2D in adulthood [18,63].

Adipocyte hypertrophy alters the balance of adipokines, leading to monocyte infiltration in the vascular wall where they are differentiated into pro-inflammatory M1-macrophages [19,64]. Under these conditions, adipose tissue releases free fatty acids, proinflammatory adipokines (leptin, resistin, tumor necrosis factor alpha (TNFα), and interleukin-6 (IL-6) into circulation, while secretion of adiponectin is reduced [19]. The unbalance between pro- and anti-inflammatory adipokines results in the generation of ROS, which increases vascular tone by inhibiting the synthesis and action of NO leading to vasoconstriction [19]. Therefore, chronic inflammation and oxidative stress are mechanisms of endothelial dysfunction in obesity [19].

Increased visceral abdominal fat is related to hypertension, the major cardiovascular risk factor associated with obesity [65]. Overweight and obese children and adolescents who remain obese with age are at increased risk of developing cardiometabolic diseases, such as T2D, hypertension, dyslipidemia, and carotid artery atherosclerosis [66]. There is a linear relationship between hypertension and obesity in White, Black, Hispanic and Asian individuals [67]. Sustained elevations in blood pressure in obese adolescents increases the risk of developing CVD when entering adulthood [68,69]. Individuals with obesity, hyperglycemia, vascular oxidative stress and inflammation are at higher risk of hypertension [70]. Hypertension in individuals with obesity seems to be the consequence of several hemodynamic, renal and neurohormonal changes caused by excess adipose tissue [71], particularly the abdominal visceral fat. In addition, excessive sodium reabsorption in the kidneys lead to increased extracellular fluid volume and elevated blood pressure, that may injure blood vessels and organs [50]. Therefore, obesity, even in children, increases the risk of having hypertension, which ultimately predisposes to vascular alterations and development of CVD in adulthood.

### 3.1. Carotid-Intima Media Thickness

Atherosclerosis is the main cause of coronary artery disease, peripheral artery disease, and ischemic stroke [72]. It is defined as a chronic inflammatory process affecting the intima and media layers decreasing the arterial lumen, and in turn, causing reduced blood flow and ischemia [72]. In the earliest stages, atherosclerosis begins as fatty streaks where the accumulation of fat-filled macrophages, termed foam cells, begin aggregating within the intima layer. The progressive accumulation of foam cells, fibrous tissue and inflammatory proteins within the intima forms an atherosclerotic plaque called atheroma [73]. This increase in blockage adversely affects the great arteries, mainly the aorta, coronary, carotid, iliac, femoral and popliteal [72]. The link between atherosclerosis and obesity is via adipokine induced inflammation, IR, and endothelial dysfunction [74]. Studies in adolescents have shown an association between obesity, hypertension and IR with the development of atherosclerosis and greater carotid intima-media thickness (cIMT) [75].

Carotid ultrasonography is a commonly used measure of subclinical atherosclerosis [76]. Several studies have evaluated the lumen–intima and media–adventitia interfaces in relation to carotid far wall histology [77,78,79]. The distance between these interfaces reflects the cIMT [80]. An increase in cIMT is considered to reflect early arterial abnormalities that ultimately result in an atherosclerotic plaque [77]. cIMT is an independent predictor of CVD and a marker of subclinical organ damage [78]. Hypertension is a major determining factor for cIMT progression [79]. Indeed, high SBP has been associated with a greater change in cIMT, which is comparable with the effects of obesity and T2D [75]. In these populations, hypertension significantly increases the risk of higher cIMT [75].

Previous literature shows an association of cardiometabolic risk factors with increased cIMT, which is influenced by high SBP, low-density lipoprotein (LDL) cholesterol, leptin, abdominal fat, chronic inflammation and lower adiponectin levels, contributing to endothelial dysfunction and progressive development of subclinical atherosclerosis [79,81]. Higher cIMT in children with obesity was associated with an increased risk of CVD in adulthood [75,82], suggesting that untreated atherosclerosis can lead to myocardial infarction and ischemic stroke [72,73]. Zhao et al. examined the relationship between cIMT and MHO in children and adolescents and found that cIMT was positively associated with body weight and CVD risk factors like elevated blood pressure, triglycerides, fasting glucose, and low HDL cholesterol. These findings demonstrated that, even without the presence of cardiometabolic risk factors, overweight and obese children have a higher risk of developing CVD [8].

### 3.2. Arterial Stiffness

Arterial stiffness is a consequence of reduced NO availability and increased endothelin-1 production (a vasoconstrictor), enhancing vascular tone [83]. Furthermore, arterial stiffness worsens due to structural changes characterized by elastin fiber fragmentation and increased type-III collagen infiltration in the artery wall (media layer); a proceeding that is more evident in central than peripheral arteries [84]. In children with obesity, the arterial diameter and compliance increase physiologically with growth and development, which can compensate arterial pressure increase, but when tension (by high blood pressure) on the aortic wall exceeds the natural adaptation point, arterial stiffness increases [54].

Although there are various methodological approaches to measure arterial stiffness, the most recognized non-invasive technique is pulse wave velocity (PWV) using applanation tonometry, which is calculated by dividing the pulse distance traveled by time across various segments in the arterial system [84]. Carotid–femoral PWV (cfPWV) is considered the gold standard measure of arterial stiffness because it has the strongest correlation with cardiovascular morbidity and mortality in adults [85], especially in those with hypertension [86]. Adolescents with T2D have higher aortic stiffness (cfPWV) compared to subjects with and without obesity [75], and with elevated blood pressure, they show higher cfPWV, regardless of obesity [15]. In young individuals, the increase in cfPWV is attributed to arterial wall distention by increased blood pressure [87]. Brachial–ankle PWV (baPWV), an estimate of systemic arterial stiffness, increases with age, hypertension, and MetS [88]. The main peripheral arterial segment in baPWV is the femoral–ankle PWV (faPWV), which measures leg arterial stiffness and is correlated with SBP, HOMA-IR and waist circumference [89].

Children and adolescents with obesity do not necessarily show differences in arterial stiffness compared to normal weight subjects. Children with obesity may have a decrease in carotid–radial PWV (crPWV) [90] due to larger peripheral arterial diameter, which indicates a vascular adaptation to accommodate a larger blood volume. However, adolescents with obesity may have higher cfPWV and crPWV compared to adolescents without obesity [91,92]. Moreover, a 5-year follow-up study showed that adolescents with obesity from 14 to 19 years of age had a 25% increase in crPWV compared to a 3% increase in normal-weight counterparts, indicating that childhood obesity has an adverse impact on arm arterial structure [91]. These findings suggest that when the natural vascular adaptation point is exceeded, they may be at a higher risk of developing CVD due to early vascular aging.

A high pulse pressure (PP) results from an increased aortic SBP (aSBP) and a reduced or unaffected diastolic blood pressure. Peripheral PP differs from aortic PP (mainly in young people) due to a SBP increase towards the periphery. This increase in PP from the central to peripheral arteries is called PP amplification and is affected by aortic stiffening and increased wave reflection from peripheral arteries [93]. Pressure waves reflected back to the aorta in peripheral sites, such as bifurcations and arterioles, are influenced by the vasomotor tone and controlled by the balance between vasodilators (e.g., NO) and vasoconstrictors (e.g., catecholamines, endothelin-1, angiotensin-II) [88]. The augmentation index (AIx) is considered a marker of left ventricular afterload and wave reflection [94], especially in young and middle-aged adults [95]. Due to pressure overload on the left ventricle, increased aortic PP, SBP and AIx are predictors of cardiovascular events and all-cause mortality in middle-aged and older adults [93]. In fact, children with obesity show higher aSBP and PP than normal weight subjects, regardless of the presence of dyslipidemia, hypertension or sedentarism [96,97]. A greater stroke volume ejection into a stiffer aorta contributes to increased aSBP and PP in children and adolescents with obesity [97], indicating a greater left ventricular overload. In young adults, the increase in adverse cardiometabolic risk factors (i.e., obesity, SBP, lipids, glucose, and insulin) are associated with decreased brachial artery distensibility or increased stiffness [98]. Obesity predisposes individuals to an increased risk of CVD and the obesity-hypertension phenotype increases this risk, accelerating an early vascular aging process. Therefore, early detection of vascular alterations and cardiometabolic risk factors is extremely important as well as interventions that have shown to be effective to improve cardiovascular health.

## 4. Effects of L-Citrulline Supplementation on Vascular and Metabolic Parameters in Obesity

L-Cit is a non-essential amino acid that is synthesized almost exclusively by the intestines and has a regulatory key role in nitrogen homeostasis [21]. Circulating L-Cit comes mainly from glutamine metabolism and endogenous synthesis of intestinal arginase, [21] and is abundantly found in watermelon (*citrullus vulgaris*) [99]. Oral ingestion of L-Cit is well tolerated [100], and safe since no toxicity or side effects in doses up to 15 g daily have been reported [101]. L-Cit has shown greater increases in plasma L-Arg than equimolar L-Arg supplementation [100,102,103] due to inhibition of intestinal and vascular arginase, preventing the catabolism of L-Arg into urea and ornithine [104] and lack of absorption and catabolism by the liver [105].

**Figure 2:**
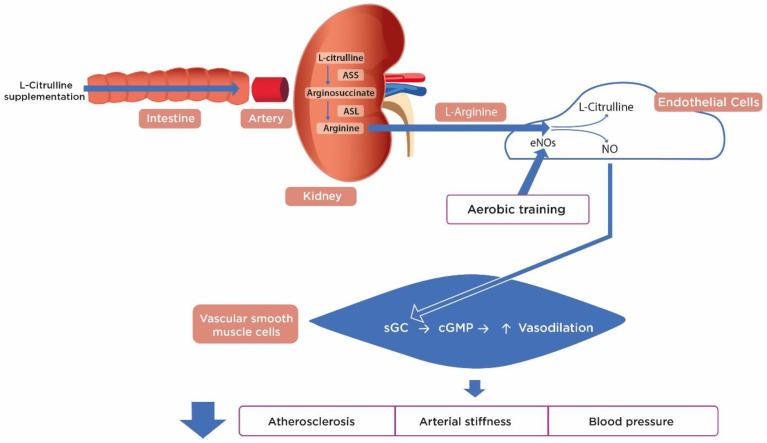
Vascular effects of aerobic training and L-Citrulline supplementation in individuals with obesity. Supplemented L-Cit is mainly metabolized in the kidneys and converted to arginosuccinate by ASS and then to L-Arg by ASL. *De novo* synthesized L-Arg is released into systemic circulation. In endothelial cells, eNOS catabolizes L-Arg to NO and L-Cit. NO diffuses into the VSMC where it stimulates sGC and subsequently activates cGMP, leading to a decrease in intracellular calcium concentrations and vasodilation. Aerobic training activates eNOS and reduces oxidative stress by increasing antioxidant capacity. Thus, L-Cit supplementation and exercise training decrease atherosclerosis, arterial stiffness, and blood pressure by improving endothelial function. L-Cit: L-Citrulline; L-Arg: L-Arginine; ASS: Arginosuccinate synthetase; ASL: Arginosuccinate lyase; eNOS: Endothelial nitric oxide synthase; NO: Nitric oxide; VSMC: Vascular smooth muscle cells; sGC: Soluble guanylyl cyclase; cGMP: Cyclic guanosine monophosphate; ↑: Increase, ↓: Decrease.

Dietary supplementation with L-Cit, either synthetic or from watermelon, increases plasma L-Arg bioavailability via *de novo* synthesis. This process occurs in the kidneys as follows: L-Cit is converted to arginosuccinate by arginosuccinate synthetase and then to L-Arg by arginosuccinate lyase. Synthesized L-Arg is then released into the renal vein and systemic circulation. In endothelial cells, eNOS catabolizes L-Arg to NO which diffuses into and induces relaxation of VSMC (vasodilation) [21,104] (Figure 2). A significant increase in plasma nitrites and nitrates (NOx) after L-Cit supplementation indicates activity of the L-Arg-NO pathway [22,26]. An indirect way to measure NO is by quantifying its metabolites as NOx, because NO is rapidly metabolized after it is produced [106].

Few studies have evaluated L-Cit supplementation in children and/or adolescents. Studies have focused on conditions in children with decreased NO. For example, mitochondrial encephalomyopathy, lactic acidosis, and stroke-like episodes (MELAS syndrome) affect many body systems, especially the brain, nervous system and muscles [103]. Children with MELAS syndrome have lower L-Cit and L-Arg flux leading to reduced NO production compared to control group [103]. In these children, L-Cit supplementation resulted in increased *de novo* L-Arg synthesis and further conversion to NO in the endothelium [103]. These findings suggest that L-Cit improves the L-Arg-NO pathway in children with MELAS syndrome.

Microvascular endothelial dysfunction is characterized by decreased release of NO in small caliber arteries and arterioles (such as those in the eyes, kidneys and nerves). This process was evaluated in children and adolescents (aged 6–17 years) with multiorgan mitochondrial disease using digital pulse wave amplitude and reactive hyperemic index (RHI) [102]. This population had lower baseline RHI than the control group, indicating microvascular endothelial dysfunction. They received similar dose of either oral L-Cit or L-Arg for 2 weeks in a cross-over design. Plasma L-Arg and L-Cit concentrations and the RHI increased 15% and 19% with both supplementations, respectively. L-Cit supplementation increased L-Arg concentrations more than L-Arg supplementation [102]. These findings suggest that short-term L-Arg and its precursor L-Cit are equally effective to reverse microvascular endothelial dysfunction. Macrovascular endothelial dysfunction occurs in arteries, such as those of the heart, brain and extremities. Morita et al. examined the effect of L-Cit on macrovascular endothelial function in adults with vasospastic angina. They reported increases in plasma L-Arg and brachial artery FMD after an 8-week intervention using a daily dose of 800 mg of L-Cit [22]. To the best of our knowledge, this is the only study that has examined the effect of L-Cit supplementation on macrovascular endothelial function in humans. L-Cit supplementation (5.8 g/day) improves macrovascular function by activation of the L-Arg-NO pathway [26]. Similarly, L-Cit supplementation (2 g/day) for 1 month increased plasma NO levels via inhibition of arginase activity in T2D patients [107]. Thus, L-Cit supplementation can be a viable therapeutic strategy to improve obesity and hyperglycemia-induced endothelial function by increasing L-Arg and NO bioavailability in middle-aged adults [22,23,26,43]. To our knowledge, there are no studies evaluating the use of L-Cit supplementation to improve vascular or metabolic parameters in children and adolescents with obesity. However, the improvements seen in circulating NOx as well as endothelial function after L-Cit supplementation in adults and in children with MELAS suggests that benefits may be useful in obese children with cardiometabolic risk factors (Table 1).

Impaired endothelial-dependent vasodilation precedes vascular dysfunction (e.g., atherosclerosis and arterial stiffness) and hypertension, which is mainly attributed to reduce NO bioavailability [37]. Thus, L-Cit supplementation has positive effects in several vascular abnormalities such as hypertension and arterial stiffness [108] by improving NO synthesis and organ blood flow and decreasing blood pressure [23,24]. A SBP reduction was evident following L-Cit supplementation (6 g/day) for 8 weeks in prehypertensive and hypertensive postmenopausal women with obesity [23,24]. However, no significant reduction in SBP was observed after 6 and 15 g/day of L-Cit for 2 weeks in overweight or obese with normal or elevated SBP, probably due to a short supplementation period and/or inefficiency of L-Cit in non-hypertensive individuals without endothelial dysfunction [109,110,111]. Therefore, L-Cit supplementation can reduce blood pressure at rest in adults with elevated blood pressure or hypertension [23,24], but not in those with normal blood pressure [22,109]. Moreover, L-Cit has demonstrated the capacity to attenuate the increase in blood pressure and arterial stiffness in young healthy overweight men [109] and patients without obesity under stressful conditions [112,113].

Endothelial dysfunction is associated with increased arterial stiffness, especially in people with hypertension [114]. Figueroa et al. found significant decreases in systemic (baPWV) and leg (faPWV) arterial stiffness after 6 g/day of L-Cit supplementation for 8 weeks in postmenopausal women with obesity and high blood pressure [25]. Similarly, Ochiai et al. found a reduction in baPWV after 7 days of supplementation with a similar dose in healthy middle-aged men with increased baPWV [26]. In contrast, baPWV was not affected by the same dose of L-Cit supplementation for 14 days in healthy men without obesity [109]. These findings suggest that L-Cit supplementation is effective in reducing peripheral arterial stiffness in patients with high arterial stiffness but not in healthy young adults. Interestingly, an inverse relationship between plasma L-Arg and baPWV was seen by Ochiai and colleagues [26], suggesting that stimulation of the L-Arg-NO pathway with L-Cit supplementation may contribute to the decrease in arterial stiffness.

Hyperinsulinemia, a common feature of IR, might enhance vasoconstriction [44]. In male C57BL/6J mice fed with a high-fat diet (HFD) and L-Cit (0.6 g/L) supplementation for 15 weeks glucose and insulin levels were reduced compared to the control group [115]. In contrast, L-Cit supplementation for 11 weeks did not affect serum glucose in both groups compared to the control group in obese/diabetic rodents. However, decreases in insulin, HOMA-IR, total cholesterol, and free fatty acids suggested improved glucose and lipid metabolism by L-Cit supplementation in these animal models [116]. In accordance, male rats supplemented with L-Cit (1 g/kg/day) had lower susceptibility to lipoprotein oxidation, indicating that L-Cit has a protective antioxidant effect [117]. Male Sprague Dawley rats received a 4-week fructose (60%) diet to develop steatosis with dyslipidemia. L-Cit (0.15 g/day) supplementation prevented hypertriglyceridemia and attenuated liver fat accumulation compared to the groups that received L-Arg or glutamine supplementation, which were ineffective. This study suggests that L-Cit acts on hepatic lipid metabolism, partially preventing hypertriglyceridemia and steatosis [118].

Physiological levels of NO stimulate glucose and fatty acid uptake and oxidation in insulin-sensitive tissues, while inhibit the synthesis of glycogen and fatty acids in target tissues and enhance lipolysis in white adipocytes [119,120,121]. Few studies have evaluated the metabolic effects of L-Cit in adult humans. L-Cit supplementation has improved serum levels of NO, lipids (triglyceride, HDL cholesterol), glucose control (insulin, glucose, glycated hemoglobin (HbA1c), HOMA-IR), and inflammation (TNF-α and C-reactive protein) in patients with obesity and T2D [107,122]. Further studies are required to evaluate the effects of L-Cit supplementation on lipid and glucose metabolism in individuals with obesity including children and adolescents.

**Table 1:** Effects of L-Citrulline supplementation on vascular and metabolic parameters in adults with obesity.

Articles	Total Sample	Intervention Group		Supplementation Characteristics			SBP (mmHg)	NOx	PWV (m/s)	Glucose (mg/dL)	Triglycerides (mg/dL)
		Sample	Age	Dose	Duration						
[23]	*n* = 41	14 women	58 ± 4 years	6 g/day	8 weeks	B	137 ± 13	28.2 ± 7.3	NM	NM	NM
						A	130 ± 15 *	35.2 ± 9.5 *			
[109]	*n* = 16	16 men	24 ± 2 (SE)	6 g/day	2 weeks	B	123 ± 3	NM	11.8 ^a^	NM	NM
						A	121 ± 3		11.2 ^a^		
[24]	*n* = 23	12 women	58 ± 1 years	6 g/day	8 weeks	B	138 ± 4	NM	NM	NM	NM
						A	131 ± 5 *^#^				
[25]	*n* = 40	14 women	58 ± 1 years	6 g/ day	8 weeks	B	137 ± 4	NM	11.5 ± 0.4 ^b^ 10.01 ± 0.2 ^c^ 14.1 ± 0.5 ^a^	NM	NM
						A	NR		11.3 ± 0.5 ^b^ 9.6 ± 0.2 ^c^* 13.2 ± 0.5 ^a^*		
[110]	*n* = 41	41 adults	18–66 years	15g/day	2 weeks	B	130 (126–134)	NM	NM	88 (84–92)	101 (77–126)
						C	−1.6 (−6.3–3.1)			NR	NR

The variables are presented as mean ± standard deviation (SD) or median (ranges). SE: standard error; *n*: total sample; NM: not measured; NR: values not reported without significant changes; B: basal measurements; A: after intervention measurements; C: post intervention change; SBP: systolic blood pressure; NOx: nitrite and nitrate; PWV: pulse wave velocity; FMD: flow-mediated dilation; ^a^ baPWV: brachial–ankle PWV; ^b^ cfPWV: carotid–femoral PWV; ^c^ faPWV: femoral–ankle PWV. Differences within groups: * *p* < 0.05, differences with the control group: ^#^ *p* < 0.05.

High fat and low muscle mass in individuals with obesity adversely affect cardiometabolic risk factors, promoting hyperglycemia, hypertension, and dyslipidemia, among others [123,124]. Evaluating body composition, male rats that received L-Cit (1 g/kg/day) supplementation decreased fat mass and increased total body lean mass, which is mainly muscle, compared with control rats [117]. Another study evaluated obese mice, where those treated with L-Cit had lower food intake and body weight than the control group. In HFD fed Sprague Dawley rats, the expression of proopiomelanocortin in the hypothalamus, a food intake suppression peptide, was significantly higher in the L-Cit group compared to the control group. Therefore, L-Cit supplementation may decrease body fat mass by appetite suppression, leading to metabolic improvements [116]. In older malnourished humans, 10 g of L-Cit supplementation for 3 weeks increased systemic amino acid availability necessary for protein synthesis in skeletal muscles. In older women, L-Cit supplementation increased total body lean mass (~1.7 kg) and decreased fat mass (~1.3 kg) [27]. More studies are required to evaluate the effects of L-Cit supplementation on body composition in children and adolescents with obesity.

## 5. Effects of the Aerobic Training in Children and Adolescents with Obesity on Vascular and Metabolic Parameters

According to the WHO, children and adolescents aged 5–17 years should do at least 60 min of moderate-to-vigorous intensity physical activity daily, most of which should be aerobic to provide health benefits [125]. Vigorous intensity activities should be incorporated at least three times per week, including those that strengthen skeletal muscles [125]. A main cause of overweight and obesity is physical inactivity [126] which results in low maximal oxygen consumption (VO_2 max_) and is associated with MetS and cardiometabolic risk factors in children and youths [127]. Physical activity yields multiple health benefits like improved vascular, metabolic and body composition parameters described in the next paragraphs. The articles included in this section, the type of training (walking or jogging [34], jump rope [31,35] and high intensity interval training (HIIT) [32,33]), intensity (moderate and high), session duration (4 to 60 min), frequency (3–5 days per week), and training duration (12–32 weeks) are described in Table 2. An increase in VO_2 peak_ [32,35] and a decrease in resting heart rate was found in children after aerobic training [31], which can be related to lower cardiometabolic risk, as elevated resting heart rate is associated with hypertension and elevated triglycerides, glucose and abdominal adiposity in children and adolescents [128].

Elevated blood pressure in children increases the risk of hypertension development during adolescence [129]. Previous studies using supra HIIT (170% peak power output) and jump rope (low-to-moderate intensity) exercises have seen decreases in SBP in prehypertensive and hypertensive children with obesity [31,32]. This reduction in SBP by 3 to 10 mmHg, respectively, is relevant to improve cardiovascular health as many newly diagnosed children with hypertension experience cardiovascular damage [130].

Aerobic exercise training has shown promising results on improving brachial artery FMD in children and adolescents. A significant improvement on FMD was evident after HIIT [32,33]. Consistent with these findings, others have reported increases in circulating NOx levels after the exercise interventions [31,32]. These findings demonstrate the beneficial effects of aerobic exercise training on improving endothelial NO production and endothelial function in obese children [131].

Boys with obesity showed a decrease in cIMT (~0.2 mm) after HIIT and supra HIIT (90% and 170% peak power output, 24 and 4 min, respectively, 3 times per week, during 12 weeks) [32]. Interestingly, meta-analytic data suggests that a longer aerobic exercise duration per week may yield small-to-moderate decreases in cIMT. This finding suggests that structural atherosclerotic changes can be reversed with regular aerobic exercise programs in obese children [32], as subclinical carotid atherosclerosis is the most common cause of CVD among children and adolescents [132].

Children and adolescents with obesity had a reduction in arterial stiffness (~0.7 to 0.8 m/s), measured with baPWV, an arterial segment that includes the aorta and leg arteries. The training protocols consisted of jump rope (at moderate-intensity for 12 weeks) [31], HIIT and supra HIIT [32]. In children and adolescents, hypertension may lead to increased arterial stiffness [54], and thus reductions in SBP may explain decreases in baPWV [31,32]. Participants had higher SBP at baseline (>120 mmHg) [31,32] than other studies that found no significant reductions in cfPWV [34,35]. Given that aerobic training at moderate- and high-intensity reduces baPWV but not cfPWV, these findings indicate a reduction in peripheral PWV.

Aerobic training at moderate-to-high intensity for 4–60 min improved at least one lipid parameter (total cholesterol [32,34], LDL cholesterol [32] triglycerides [32,33] or HDL cholesterol [35]) in obese children and adolescents. In a systematic review, the magnitude of the effects of aerobic training on the metabolic profile of obese children was associated with the intensity (≤75% heart rate max) and duration (60 min) and frequency (3 times a week) of the exercise session [133]. These findings suggest that high-intensity aerobic exercise sessions could be efficient in improving lipid profile in obese children and adolescents. However, diet must be monitored to yield such improvements.

Aerobic training shows a favorable impact on body composition in children and adolescents with obesity, of which had a reduction in waist circumference [31,33], total body fat percentage [31,35], and increases in lean mass [31] after aerobic training at moderate-to-high intensity for 12–32 weeks. One study shows that waist circumference is associated with aerobic capacity (an important health-related factor) in boys and girls [134]. These studies suggest that aerobic training is fundamental to achieve beneficial changes in fat and muscle mass, thus, a lower cardiovascular risk in children with obesity.

Exercise training in children and adolescents with obesity showed positive effects on cardiometabolic risk factors (Table 3). It is important to emphasize that exercise recommendations for children are designed for the prevention of cardiometabolic risk factors as well as depression and anxiety [125]. Therefore, aerobic training should be included in CVD prevention and treatment programs in children and adolescents with obesity.

## 6. Effects of L-Citrulline Supplementation and Exercise Training in Individuals with Obesity on Vascular and Metabolic Parameters

**Figure 3:**
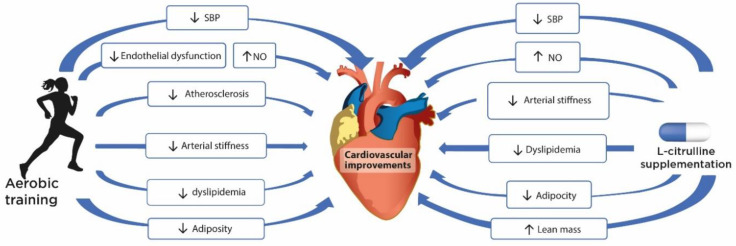
Cardiovascular improvements with L-Citrulline supplementation and moderate-to-high intensity aerobic training. In children and adolescents with obesity and cardiometabolic alterations, aerobic training helps to improve blood pressure (SBP), endothelial function (NO and FMD), atherosclerosis (cIMT), arterial stiffness (baPWV), dyslipidemia (triglycerides, total cholesterol, LDL-C and HDL-C), and body composition (waist circumference and total body fat). L-Cit supplementation in adults with cardiometabolic alterations helps to improve NO production, blood pressure (SBP), arterial stiffness (baPWV and faPWV), dyslipidemia (triglycerides and HDL-C), glucose control (insulin, glucose and HOMA-IR) and body composition (total body fat and lean mass). L-Cit: L-Citrulline; SBP: systolic blood pressure; NO: nitric oxide; FMD: flow-mediated dilation; cIMT: intima–media thickness; baPWV: brachial–ankle PWV; LDL-C: LDL cholesterol; HDL-C: HDL cholesterol; faPWV: femoral–ankle PWV; HOMA-IR: homeostatic model assessment-insulin resistance; ↑: Increase; ↓: Decrease.

The interest on the potential synergistic or additive effect of L-Cit supplementation and exercise training is supported by improvements in vascular and metabolic parameters. Aerobic training improves body composition, lipid profile, endothelial function, atherosclerosis, arterial stiffness, and blood pressure in children and adolescents with obesity [31,32,33,34,35]. L-Cit supplementation increases the bioavailability of L-Arg and NO to enhance vasodilation [22,23,26], improve arterial stiffness, and regulate blood pressure by having an antihypertensive effect in adults [23,24], and may improve endothelial function in children and adolescents with obesity with lower NO production and endothelial dysfunction [102,103]. L-Cit supplementation has also demonstrated to improve glucose control [107,122], muscle protein synthesis [27], and body composition (lean mass and fat mass) [116] (Figure 3). The combination of aerobic exercise training and L-Cit supplementation on metabolic and cardiovascular parameters in obesity has only been studied in adults (Table 4). The most important benefits are discussed below.

Hypertension is the most important risk factor for CVD in adults. Effective interventions are constantly searched to improve blood pressure. Obese postmenopausal women received L-Cit (6 g/day), whole-body vibration training (an alternative strength training modality) or the combination of both interventions for 8 weeks [23]. All groups similarly decreased SBP, indicating that this combined intervention has no additive effect on blood pressure at the prescribed dose and duration. However, the combined intervention was more effective than each individual intervention to decrease pressure wave reflection and aortic stiffness, which may be clinically significant for postmenopausal women with elevated blood pressure and hypertension [23,25]. These findings showed that the combination of exercise training (WBVT) and L-Cit supplementation have beneficial effects on arterial function in obese postmenopausal women [23,25]. This population is of special interest because they have higher risk of CVD attributed to aging and sex-related increases in aSBP, wave reflection, and proximal aortic stiffness [135]. However, these studies do not represent the entire population of adults with obesity, and WBVT is not a conventional strength training modality.

There are no studies evaluating the metabolic effects of combined exercise training and L-Cit in individuals with obesity. In a study by Buckinx et al., two groups of healthy older adults without obesity combined HIIT with L-Cit supplementation (10 g/day) or placebo for 12 weeks with no impact on metabolic markers (glucose, insulin, HOMA-IR or lipid profile) [136], as they had no basal metabolic abnormalities. Given that the cardiometabolic effects of oral L-Cit might be via *de novo* L-Arg synthesis, L-Cit and L-Arg supplementation may produce similar metabolic effects. In a randomized placebo-controlled trial, two groups of middle-aged adults with obesity and T2D received a low-calorie diet and exercise training (both aerobic and resistance) combined with either L-Arg supplementation (8.3 g) or placebo for 3 weeks. Compared with the placebo group, L-Arg supplementation had an additive effect on vascular function, glucose and lipid metabolism, and fat mass as well as prevented the loss of muscle mass associated with hypocaloric diet [137]. Since oral L-Cit is more efficient than L-Arg for increasing plasma L-Arg availability, L-Cit supplementation combined with exercise training may have similar or possibly greater beneficial effects on vascular and metabolic function than those observed with L-Arg supplementation. However, clinical trials are needed to validate this hypothesis.

Several studies have evaluated the effect of exercise training and L-Cit supplementation on anthropometric parameters, finding no significant changes in BMI [23,25,136,138]. Buckinx et al. studied older adults with obesity and muscle weakness who performed HIIT with L-Cit supplementation (10 g/day) or placebo for 12 weeks. Although there were no significant increases in lean mass with HIIT and L-Cit supplementation, a decrease in total and leg fat mass percentages were noted after HIIT and L-Cit supplementation [138]. Despite the lack of impact on muscle mass, HIIT with L-Cit improved arm muscle strength and walking speed [138], suggesting that the combination of L-Cit and HIIT may improve total body fat loss and increase strength more than exercise training alone.

A research group examined the effects of strength training (WBVT) combined with L-Cit or placebo on body composition and muscle strength in obese postmenopausal women. It was found that WBVT alone and WBVT plus L-Cit supplementation had similar significant decreases in body fat percentage [25]. L-Cit supplementation and WBVT favored the increase in leg lean mass compared with the WBVT and placebo groups. However, the improvements in leg muscle strength were similar with or without L-Cit, indicating no additional benefit of L-Cit in postmenopausal women with obesity. Although L-Cit combined with unconventional strength training had additive positive effects on arterial stiffness and muscle mass in obese postmenopausal women [25], the impact on endothelial function as a potential mechanism was not examined. These findings suggest that both types of training (HIIT and WBVT) combined with L-Cit supplementation have beneficial effects on body composition.

To our knowledge, only the studies reviewed in this section have reported effects on vascular and muscular parameters using L-Cit in combination with different types of exercise training in adults with obesity. There are no studies evaluating changes in glucose metabolism or lipid profile in individuals with obesity using L-Cit plus exercise training. As a limitation, in the reviewed studies, the individuals without obesity did not have metabolic alterations to evaluate the effect of these combined interventions. Another limitation is that L-Cit has not been evaluated in combination with resistance training. Unfortunately, the combination of L-Cit plus aerobic training has not been examined in children and adolescents. The studies that investigated L-Cit supplementation in children focused on improving a specific condition, such as MELAS. Randomized, placebo-controlled studies are required to evaluate the combination of these two interventions in children and adolescents with obesity because results of previous studies suggest that L-Cit and exercise training can be an effective strategy to counteract the effects of obesity on vascular and metabolic function at early stages of life.

**Table 4:** Effects of L-Citrulline supplementation and exercise training on vascular and metabolic parameters in adults with obesity.

Article	Total Sample	Intervention Groups		Exercise Characteristics			L-Citrulline Dose		SBP (mmHg)	aSBP (mmHg)	Aix (%)	NOx (µmol/L)	PWV (m/s)	WC (cm)
		Sample	Age	Training	Session	Frequency/ Duration								
[23]	*n* = 41	13 women	58 ± 3 years	WBVT: dynamic leg exercises	1–5 sets (30–60 s)	3 days/week For 8 weeks	6 g/day	B	140 ± 9	133 ± 9	43.5 ± 10.2	28.2 ± 14.9	NM	NM
								A	132 ± 9 *	123± 9 *	33.3 ± 8.3 *	38.3 ± 19.6 *		NM
[25]	*n* = 41	13 women	58 ± 1 years	WBVT: dynamic leg exercises	1–5 sets (30–60 s)	3 days/week For 8 weeks	6 g/day	B	140 ± 3	NM	NM	NM	11.7 ± 0.3 ^b^ 14.7 ± 0.4 ^a^ 10.4 ± 0.3 ^c^	NM
								A	NR				10.8± 0.3 ^b^* 13.4 ± 0.4 ^a^* 9.8 ± 0.2 ^c^*	NM
[138]	*n* = 56	26 adults	65.7 ± 4.2 years	HIIT	30 min	3 days/week For 12 weeks	10 g/day	B	NM	NM	NM	NM	NM	107 ± 11
								A						104 ± 11 *
[136]	*n* = 44	23 adults	67.6 ± 5.01 years	HIIT	30 min	3 days/week For 12 weeks	10 g/day	B	NM	NM	NM	NM	NM	98.3 ± 10.2
								A						95.9 ± 10.9 *

The variables are presented as mean ± standard deviation (SD). *n*: total sample; NM: not measured; NR: values not reported; B: basal measurements; A: after intervention measurements; BMI: body mass index; WC: waist circumference; SBP: systolic blood pressure; aSBP: aortic systolic blood pressure; PWV: pulse wave velocity; ^a^ baPWV: brachial–ankle PWV; ^b^ cfPWV: carotid–femoral PWV; ^c^ faPWV: femoral–ankle PWV; AIx: augmentation index; NOx: nitrite and nitrate; WBVT: whole-body vibration training. Differences within groups: * *p* < 0.05.

## 7. Conclusions and Remarks

Children and adolescents with obesity may have early vascular aging characterized by endothelial dysfunction, elevated blood pressure, arterial stiffness, and multiple cardiometabolic risk factors that increases the risk of CVD development in adulthood. Aerobic training is essential to reduce cardiometabolic disorders associated with obesity in children and adolescents. Interventions at moderate-to-high intensity for 12–32 weeks have showed positive effects on endothelial function, arterial stiffness, atherosclerosis, blood pressure, and IR. L-Cit supplementation increases the bioavailability of L-Arg and NO in children and adults. In adults, L-Cit supplementation has shown to be effective for improving blood pressure, arterial stiffness, body composition (lean and fat mass), and metabolism (glucose and lipid profile), especially in obese individuals. The WBVT and L-Cit supplementation is an effective strategy to improve cardiovascular and muscular health in adults with obesity; thus, further studies are needed to test the effectiveness of this combination in children and young adults with obesity and cardiometabolic alterations.

## Figures and Tables

**Table 2:** Characteristics of children and adolescents with obesity and aerobic training.

Article	Total Sample	Group	Intervention Group					Exercise Training Characteristics				
			Sample	Female	Male	Age (Years)	Body Mass Index (kg/m^2^)	Training	Intensity	Session (Minutes)	Frequency (Days/Week)	Duration (Weeks)
[35]	175	Exercise	90	55	35	9.7 (9.5, 9.8)	25.9 (25.0, 26.9)	Jump rope	HR >140 bpm	40	5	32
		Control	85	47	38	9.7 (9.5, 9.9)	25 (24.4, 26.8)	N/A	N/A	N/A	N/A	N/A
[32]	48	HIIT	11	0	11	11± 0.3	24.2 ± 1.0	HIIT: 8 × 2 min intervals	90% PPO	24	3	12
		Supra-HIIT	15	0	15	11± 0.2	26.5 ± 0.9	Supra HIIT: 8 × 20 s intervals	170% PPO	4	3	12
		Control	11	0	11	10.6 ± 0.3	53.6 ± 4.0	N/A	N/A	N/A	N/A	N/A
[34]	118	Aerobic exercise	38	13	25	14.4 ± 1.6	>85th percentile	Walking/jogging	60–65% VO_2_	40–60	3	24
[31]	40	Exercise	20	20	0	15 ± 1	26 ± 3	Jump rope	40–70% HRR	50	5	12
		Control	20	20	0	15 ± 1	25 ± 2	N/A	N/A	N/A	N/A	N/A
[33]	38	Exercise	25	13	12	15.1 ± 1	28 ± 3	HIIT: 2–6 (100 m running sprints)	NP	10–22	3	12

The variables are presented as mean ± standard deviation (SD) or median (ranges). N/A: not applicable; NM: not measured, instead obesity was considered ≥ 25.0% for men and ≥ 35.0% for women; MICT: moderate-intensity continuous training; HIIT: high-intensity interval training; bpm; beat/minute; HR: heart rate; HRR: heart rate reserve; HR max: maximum heart rate; PPO: peak power output; VO_2:_ maximal oxygen consumption; NP: no prescription.

**Table 3:** Effects of exercise training on cardiovascular disease risk factors in children and adolescents with obesity.

Articles	Group	SBP (mmHg)		FMD (%)		cIMT (mm)		PWV (m/s)		SBP (mmHg)		LDL-C (mg/dL)	
		Baseline	Post/ Mean Changes	Baseline	Post/ Mean Changes	Baseline	Post/ Mean Changes	Baseline	Post/ Mean Changes	Baseline	Post/ Mean Changes	Baseline	Post/ Mean Changes
[35]	Exercise	105 (103, 107)	0.1 (−1.5, 1.7)	NM		105 (103, 107)	0.1 (−1.5, 1.7)	5.1 (4.9, 5.3) ^b^	−0.02 (−0.23, 0.2)	105 (103,107)	0.1 (−1.5, 1.7)	102 (95,110)	−6 (−10, −2)
	Control	102 (100, 104)	0.3 (−1.3, 2)	NM		102 (100, 104)	0.3 (−1.3, 2)	5.1 (4.9, 5.2) ^b^	−0.04 (−0.18, 0.26)	102 (100,104)	0.3 (−1.3, 2)	103 (96,110)	−5 (−9, −1)
[32]	HIIT	128 ± 4	125 ± 4	8.9	11.1 *^#^	128 ± 4	125 ± 4	9.97 ^a^	9.3 *^#^	128 ± 4	125 ± 4	112 ± 6	88 ± 6 *
	Supra HIIT	127 ± 4	116 ± 3 *	7.9	10.1 *	127 ± 4	116 ± 3 *	9.86 ^a^	9.06 *^#^	127 ± 4	116 ± 3 *	105 ± 6	81 ± 5 *^#^
	Control	121 ± 4	121 ± 4	8.3	7.4	121 ± 4	121 ± 4	10.04 ^a^	10.2	121 ± 4	121 ± 4	111 ± 7	104 ± 6
[34]	Aerobic exercise	112 ± 8	0.9 ± 1.4	NM		112 ± 8	0.9 ± 1.4	5.97 ± 0.75 ^b^	−0.19 ± 0.14	112 ± 8	0.9 ± 1.4	87 ± 24	−4 ± 2
[31]	Exercise	126 ± 3	120 ± 2 *^#^	NM		126 ± 3	120 ± 2 *^#^	8.2 ± 1 ^a^	7.4 ± 0.2 *^,#^	126 ± 3	120 ± 2 *^#^	NM	
	Control	126 ± 4	127 ± 5.3	NM		126 ± 4	127 ± 5.3	8.2 ± 0.5 ^a^	8.1 ± 0.2	126 ± 4	127 ± 5.3	NM	
[33]	Exercise	NM		7.9	12.2 *	NM		NM		NM		90 ± 23	99 ± 25

This table describes the effects of exercise training on cardiometabolic disease risk factors of the studies shown in Table 2. The variables are presented as mean ± SD or median (ranges). NM: not measured; WC: waist circumference; SBP: brachial systolic blood pressure; cIMT: intima-media thickness; PWV: pulse wave velocity; ^a^ baPWV: brachial–ankle PWV; ^b^ cfPWV: carotid–femoral PWV; FMD: flow-mediated dilation; LDL-C: low density lipoprotein cholesterol; HIIT: high-intensity interval training. Differences within groups: * *p* < 0.05, differences between groups: ^#^ *p* < 0.05.

## Data Availability

No applicable.
